# Noncoding RNAs Associated with Therapeutic Resistance in Pancreatic Cancer

**DOI:** 10.3390/biomedicines9030263

**Published:** 2021-03-07

**Authors:** Seung Wan Son, Mun Gyu Song, Ba Da Yun, Jong Kook Park

**Affiliations:** Department of Biomedical Science, Hallym University, Chunchon 24252, Korea; miyanae@naver.com (S.W.S.); smgdd@naver.com (M.G.S.); asalama@naver.com (B.D.Y.)

**Keywords:** noncoding RNA, microRNA, long noncoding RNA, circular RNA, therapeutic resistance, pancreatic cancer

## Abstract

Therapeutic resistance is an inevitable impediment towards effective cancer therapies. Evidence accumulated has shown that the signaling pathways and related factors are fundamentally responsible for therapeutic resistance via regulating diverse cellular events, such as epithelial-to-mesenchymal transition (EMT), stemness, cell survival/apoptosis, autophagy, etcetera. Noncoding RNAs (ncRNAs) have been identified as essential cellular components in gene regulation. The expression of ncRNAs is altered in cancer, and dysregulated ncRNAs participate in gene regulatory networks in pathological contexts. An in-depth understanding of molecular mechanisms underlying the modulation of therapeutic resistance is required to refine therapeutic benefits. This review presents an overview of the recent evidence concerning the role of human ncRNAs in therapeutic resistance, together with the feasibility of ncRNAs as therapeutic targets in pancreatic cancer.

## 1. Introduction

Most pancreatic cancer (PaC) patients are diagnosed at an advanced stage owing to the lack of early detections; therefore, surgical management is unavailable for over 80% of patients [1,2]. Moreover, PaC is resistant to treatment options, such as radiotherapy, chemotherapy, and targeted therapy [1,3,4]. These features underline the requirement of developing more effective treatments for PaC. Noncoding RNAs (ncRNAs) are differentially expressed in cancer and control diverse signaling pathways involved in the regulation of therapeutic resistance [5,6,7,8]. An improved understanding of the relationship between therapeutic resistance and ncRNAs can provide meaningful insights to develop new treatment strategies for PaC. This review highlights the role of human ncRNAs in modulating the effectiveness of treatments in PaC.

### 1.1. Noncoding RNAs

A large number of studies have provided evidence that microRNAs (miRNAs), in general, repress the translation and induce the degradation of their target messenger RNAs (mRNAs) via binding to the 3′ untranslated region (3′ UTR) [9]. Long noncoding RNAs (lncRNAs) play critical roles in gene regulation [10]. They can regulate chromatin structure, gene transcription, and pre-mRNA splicing [11]. Furthermore, the stability of proteins is affected by lncRNAs [12]. Another functional competency of lncRNAs is to sponge miRNAs, thus constraining the abundance and activity of miRNAs. For example, a recent study demonstrated that lncRNA-ADPGK-AS1 inhibits miR-205-5p, thereby promoting the progression of PaC via activating epithelial-to-mesenchymal transition (EMT) [13]. Moreover, circular RNAs (circRNAs) can control gene transcription via interaction with RNA-binding proteins [8,14]. They also regulate the signaling pathways through the sequestration of miRNAs [8,15].

### 1.2. Mechanisms of Therapeutic Resistance

Therapeutic resistance is related to EMT, cancer stem cells (CSCs), and efflux transporters. PaC cells expressing high levels of EMT markers are resistant to gemcitabine, 5-fluorouracil (5-FU), and cisplatin. In fact, the efficacy of these anti-cancer agents is restored by an inhibition of zinc finger E-box-binding homeobox (*ZEB1*) [16,17,18]. Another study also showed that maintenance of the EMT program mediates radioresistance in PaC [19]. In addition, pancreatic CSCs are resistant to currently available therapies owing to their hallmarks, including the intense expression of anti-apoptotic factors and drug efflux transporters [20]. The treatment of gemcitabine promotes cancer stemness, thus reinforcing chemoresistance in PaC [21]. Thus, the inhibition of cancer stemness has been attempted to increase therapeutic efficacy against PaC [22,23]. In particular, cancer growth and metastasis are remarkably suppressed by the combination of gemcitabine with afatinib, a cancer stemness inhibitor [23].

Moreover, cellular factors related to survival and apoptosis are linked to therapeutic resistance. A recent study showed that gemcitabine resistance is aggravated by an activation of AKT serine/threonine kinase (AKT) signaling; therefore, AKT inhibition augments the efficacy of gemcitabine by activating apoptotic cell death in vitro and in vivo [24]. In addition, extracellular signal-regulated kinase (ERK) positively regulates the level of anti-apoptosis factors such as B-cell CLL/lymphoma 2 (*BCL2*), impeding caspase activations [25]. Activated ERK is involved in therapeutic resistance to several agents, such as gemcitabine, paclitaxel, and 5-FU [26,27,28].

Accumulating evidence has shown that autophagy has a cytoprotective activity against anti-cancer therapies [29,30]. In PaC, the sensitivity of cells to doxorubicin is enhanced by the pharmacological suppression of autophagy [31]. The silencing of autophagy-related 5 (*ATG5*) increases doxorubicin-induced apoptosis as well [31]. In addition, autophagy is induced by several agents, including gemcitabine, 5-FU, and salinomycin. The inhibition of autophagy augments the cytotoxicity of these agents in PaC [32,33,34]. It suggests that cancer cells withstand stressful conditions via the compensatory activation of autophagy.

## 2. Oncogenic miRNAs Conferring Therapeutic Resistance

### 2.1. EMT-Regulating MiRNAs

#### 2.1.1. MiR-10a-5p

**Figure 1:**
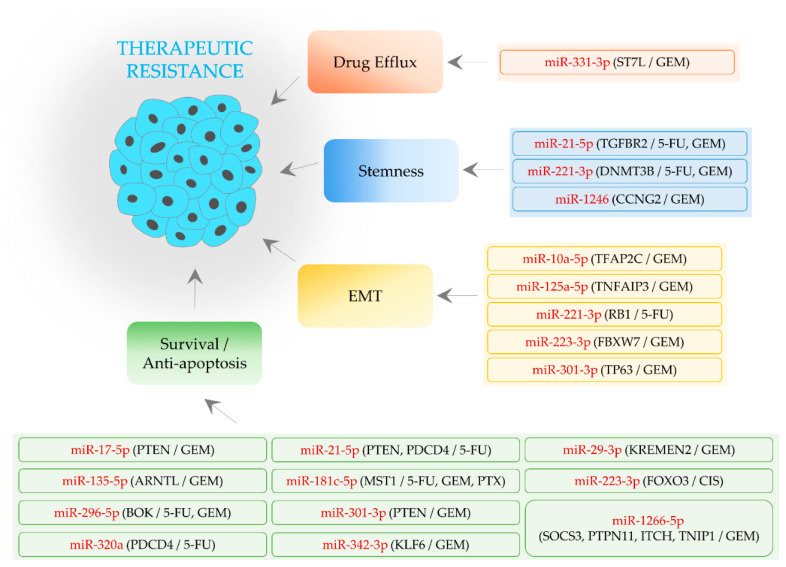
MiRNA-mediated aggravation of therapeutic resistance in pancreatic cancer (PaC). Oncogenic miRNAs in rounded rectangles are shown in red. Round brackets denote target genes of miRNAs and then therapeutic agents affected by miRNAs. Activation is indicated by an arrow. GEM: gemcitabine; 5-FU: 5-fluorouracil; PTX: paclitaxel; CIS: cisplatin.

It has been reported that miR-10a-5p can act as a tumor-suppressive miRNA or an oncogenic miRNA, depending on cancer types. The overexpression of miR-10a-5p suppresses cell cycle progression and metastasis in cervical and colorectal cancer, respectively [35,36]. By contrast, a recent study demonstrated that miR-10a-5p confers gemcitabine resistance by targeting transcription factor-activating enhancer-binding protein 2C (*TFAP2C*) in PaC [37]. In this study, it was observed that the overexpression of miR-10a-5p or TFAP2C increases or decreases the expression of EMT-related genes such as snail family transcriptional repressor 1 (*SNAI1*), respectively (Figure 1 and Table 1). In line with this, the administration of gemcitabine inefficiently reduces the growth of miR-10a-5p-overexpressing PaC cells in a mouse xenograft model [37]. However, another study showed that TFAP2C triggers tumorigenesis and EMT by upregulating the level of transforming growth factor-β receptor 1 (*TGFBR1*) in lung cancer [38]. These findings suggest that the function of TFAP2C is dissimilar in a cellular context-dependent manner.

#### 2.1.2. MiR-125a-5p

Several studies demonstrated that tumor necrosis factor alpha-induced protein 3 (*TNFAIP3*, also known as *A20*) inhibits EMT. The knockdown of TNFAIP3 facilitates the migration and invasion of nasopharyngeal cancer cells [61]. Furthermore, TNF-induced motility is suppressed by TNFAIP3 in hepatocellular carcinoma cells [62]. Moreover, TNFAIP3 diminishes the level of EMT markers such as ZEB1 via inactivating nuclear factor kappa B (NF-κB) signaling, thereby negatively modulating the migration and invasion capacities of lung cancer cells [63]. These results suggest that miRNAs targeting TNFAIP3 can regulate the sensitivity of cells to anti-cancer therapies. In PaC, it was found that TNFAIP3 is targeted by miR-125a-5p and that both miR-125a-5p overexpression and TNFAIP3 knockdown desensitize cells to gemcitabine [43] (Figure 1 and Table 1).

#### 2.1.3. MiR-221-3p

It has been noticed that miR-221-3p facilitates EMT and therapeutic resistance in several types of cancer. For instance, miR-221-3p is transcriptionally activated by Twist family BHLH transcription factor 2 (*TWIST2*) and enhances cell migration, invasion, and lymphatic metastasis in cervical cancer [64]. Additionally, miR-221-3p mediates doxorubicin resistance in breast cancer cells [65]. In PaC, miR-221-3p can promote EMT by targeting RB transcriptional corepressor 1 (*RB1*), thereby desensitizing cells to 5-FU [47] (Figure 1 and Table 1).

#### 2.1.4. MiR-223-3p

It was demonstrated that miR-223-3p is upregulated in gemcitabine-resistant PaC cells [49,50]. Further evidence has shown that miR-223-3p is capable of targeting F-box and WD repeat domain-containing 7 (*FBXW7*) and induces gemcitabine resistance via activating Notch signaling-mediated EMT [49,50] (Figure 1 and Table 1). Moreover, it was revealed that the level of miR-223-3p is downregulated by genistein and that the combination of genistein and miR-223-3p inhibitors synergistically sensitizes resistant cells to gemcitabine in vitro and in vivo [50]. However, miR-223-3p can repress the migration and invasion of osteosarcoma cells [66], implying that the role of miR-223-3p is disparate in a cell-type-dependent manner.

#### 2.1.5. MiR-301-3p

Several studies showed that miR-301-3p is highly expressed in various cancers and prompts migration, invasion, and EMT process [67,68,69]. In PaC, EMT is also promoted by miR-301-3p, which directly targets tumor protein p63 (*TP63*) [53]. In their study, it was noticed that gemcitabine resistance is induced by miR-301-3p overexpression (Figure 1 and Table 1). Besides, since the transcription of miR-301-3p is activated by hypoxia [54], the miR-301-3p/TP63 axis may contribute to gemcitabine resistance under hypoxic conditions.

### 2.2. Stemness-Regulating MiRNAs

#### 2.2.1. MiR-21-5p and MiR-221-3p

Cancer stemness is enhanced by miR-21-5p, which is capable of targeting TGFBR2 in colorectal cancer. Furthermore, it was identified that miR-221-3p intensifies cancer stemness by targeting DNA methyltransferase-3 beta (*DNMT3B*) in breast cancer [70,71]. Both miRNAs are upregulated in stem-like PaC cells compared to non-stem cancer cells [40], suggesting that these miRNAs can play an essential role in stemness regulation, probably via targeting TGFBR2 and DNMT3B in PaC. Notably, the knockdown of miR-21-5p and miR-221-3p suppresses the population of stem-like PaC cells, as well as increasing the effects of 5-FU and gemcitabine in vitro. Moreover, the in vivo growth of stem-like PaC cells is significantly reduced by the combined knockdown of miR-21-5p and miR-221-3p [40]. These results suggest that the inhibition of these miRNAs can be a potential therapeutic strategy for PaC (Figure 1 and Table 1).

#### 2.2.2. MiR-1246

Microarray analysis of miRNA expression showed that miR-1246 is one of the miRNAs upregulated in gemcitabine-resistant PaC cells. Further analyses exhibited that miR-1246 strengthens the sphere-forming capacity of cells by targeting cyclin G2 (*CCNG2*) [59], which negatively regulates cancer stemness via inactivating Wingless (Wnt)/β-catenin signaling [72]. It was also confirmed that the knockdown of miR-1246 re-sensitizes resistant cells to gemcitabine [59] (Figure 1 and Table 1).

### 2.3. Cell Survival- and Apoptosis-Regulating MiRNAs

#### 2.3.1. MiR-17-5p, MiR-21-5p, MiR-301-3p, and MiR-320a

A number of studies presented that miRNAs promote therapeutic resistance via targeting phosphatase and tensin homolog (*PTEN*) in PaC. Both miR-17-5p and miR-301-3p contribute to gemcitabine resistance by targeting PTEN [39,55]. In the case of miR-17-5p, the expression of this miRNA is increased by GDNF family receptor alpha-2 (*GFRA2*), connoting that GFRA2 can develop gemcitabine resistance via the miR-17-5p/PTEN axis [39]. Besides, the combination of miR-301-3p inhibitors with gemcitabine significantly inhibits the in vivo growth of gemcitabine-resistant cells [55]. PTEN is also targeted by miR-21-5p [41], which modulates cancer stemness as well (Section 2.2.1). Additionally, miR-21-5p can directly target programmed cell death 4 (*PDCD4*), hence advancing 5-FU resistance [41]. Moreover, miR-320a was confirmed to regulate PDCD4, thus promoting 5-FU resistance [56] (Figure 1 and Table 1).

#### 2.3.2. MiR-29-3p

Wnt/β-catenin signaling is activated in cancer and controls numerous events, such as apoptosis and therapeutic resistance. The inhibition of Wnt/β-catenin signaling induces apoptosis and reverses gemcitabine resistance in PaC [73,74] (also see Section 2.2.2 about Wnt/β-catenin-stemness connections). In addition, the inhibition of Wnt receptors by vantictumab retards the growth of PaC and enhances the anti-cancer activity of paclitaxel [75,76]. Concerning miRNAs, miR-29-3p was suggested to target several Wnt signaling antagonists, including kringle-containing transmembrane protein 2 (*KREMEN2*), therefore activating Wnt/β-catenin signaling and abrogating gemcitabine-induced apoptosis in PaC cells [42] (Figure 1 and Table 1). However, miR-29-3p functions as an anti-metastatic factor in gastric cancer cells [77], suggesting that more investigation on the role of miR-29-3p is warranted.

#### 2.3.3. MiR-135-5p

Aryl hydrocarbon receptor nuclear translocator-like (*ARNTL*, also named *BMAL1*) is expressed at low levels in PaC tissues [78]. Experimental evidence showed that ARNTL overexpression positively regulates apoptosis by stimulating the tumor protein p53 (*TP53*) pathway [78]. Lately, it was presented that miR-135-5p aggravates gemcitabine resistance by targeting ARNTL in PaC. The suppression of miR-135-5p augments gemcitabine-induced apoptosis, along with caspase-3 activations in vitro. In mouse xenograft models of PaC, the overexpression and downregulation of miR-135-5p desensitize and sensitize cells to gemcitabine, respectively [44] (Figure 1 and Table 1).

#### 2.3.4. MiR-181c-5p

The induction of apoptosis can be impeded by miR-181c-5p owing to its potentiality to target Fas cell surface death receptor (*FAS*) in Ewing’s sarcoma [79]. Moreover, it was unveiled that miR-181c-5p renders PaC cells resistant to gemcitabine, 5-FU, and paclitaxel by reducing the level of drug-induced apoptosis [45]. In this study, the Hippo signaling pathway was found to be restrained by miR-181c-5p, which targets multiple genes such as mammalian STE20-like protein kinase 1 (*MST1*) [45] (Figure 1 and Table 1). The Hippo pathway has been proven to inactivate Yes-associated protein 1 (*YAP1*), resulting in the downregulation of anti-apoptotic factors such as BCL2 [80,81]. However, miR-181c-5p can inhibit tumorigenesis and stemness in cervical cancer and glioblastoma [82,83], pointing out that the function of miR-181c-5p is highly diverse depending on the cancer type.

#### 2.3.5. MiR-223-3p

As stated in Section 2.1.4, miR-223-3p has a resistance-promoting activity by regulating EMT. Further, miR-223-3p can drop the sensitivity of PaC cells to cisplatin by directly repressing forkhead box O3 (*FOXO3*), a pro-apoptotic factor [51]. The silencing of miR-223-3p increases cisplatin-induced apoptosis, along with an upregulation of FOXO3 expression [51] (Figure 1 and Table 1).

#### 2.3.6. MiR-296-5p

BCL2-related ovarian killer (*BOK*) is a non-canonical member of the BCL2 family and serves as a tumor suppressor by triggering cell death [84,85]. A recent study unveiled that the overexpression of miR-296-5p contributes to resistance to 5-FU and gemcitabine by directly targeting BOK [52] (Figure 1 and Table 1). In addition, miR-296-5p enhances the invasion and EMT process, suggesting that miR-296-5p can act as an EMT-stimulating miRNA [52].

#### 2.3.7. MiR-342-3p

Leptin has been reported to prompt cell proliferation and survival via activating phosphoinositide 3-kinase (PI3K)/AKT signaling [86,87]. Moreover, leptin can activate NF-κB, leading to therapeutic resistance [88]. A further study on the mechanism underlying leptin-mediated drug resistance revealed that the treatment of PaC cells with leptin increases the level of miR-342-3p, which targets Kruppel-like factor 6 (*KLF6*) [58], an apoptosis-inducer [89,90]. In addition, treatments with miR-342-3p inhibitors ameliorate gemcitabine resistance by increasing apoptosis in vitro. Further, it was observed that miR-342-3p inhibitors in combination with gemcitabine improve survival in a xenograft mouse model of PaC [58] (Figure 1 and Table 1).

#### 2.3.8. MiR-1266-5p

The activation of NF-κB and signal transducer and activator of transcription 3 (*STAT3*) blocks apoptotic cell death by upregulating the expression of pro-survival factors such as BCL2-like 1 (*BCL2L1*, also called *BCL-XL*) [91,92]. Particularly, both NF-κB and STAT3 signaling can potentiate gemcitabine resistance in PaC [93,94]. Recent evidence has shown that miR-1266-5p activates the NF-κB and STAT3 pathways by targeting diverse genes, namely suppressor of cytokine signaling 3 (*SOCS3*), protein tyrosine phosphatase non-receptor type 11 (*PTPN11*), itchy E3 ubiquitin-protein ligase (*ITCH*), and TNFAIP3-interacting protein 1 (*TNIP1*) [60]. Indeed, the susceptibility of PaC cells to gemcitabine is restored by miR-1266-5p silencing. Notably, the inhibition of miR-1266-5p improves the gemcitabine-mediated suppression of PaC growth together with caspase-3 activations in vivo [60] (Figure 1 and Table 1).

### 2.4. An MiRNA Associated with Drug Efflux

#### MiR-331-3p

Wnt/β-catenin signaling can provoke multidrug resistance via upregulating the level of drug transporters, such as ATP-binding cassette subfamily B member 1 (*ABCB1*), ABCC1, and ABCG2 [95,96,97,98,99]. It was recently demonstrated that gemcitabine resistance is promoted by miR-331-3p in PaC. This miRNA activates Wnt/β-catenin signaling by inhibiting its target gene, suppression of tumorigenicity 7-like (*ST7L*), thus leading to an increase in ABCB1, ABCC1, and ABCG2 levels [57] (Figure 1 and Table 1).

## 3. Tumor-Suppressive MiRNAs Alleviating Therapeutic Resistance

### 3.1. EMT-Regulating MiRNAs

#### 3.1.1. MiR-30a-5p

**Figure 2:**
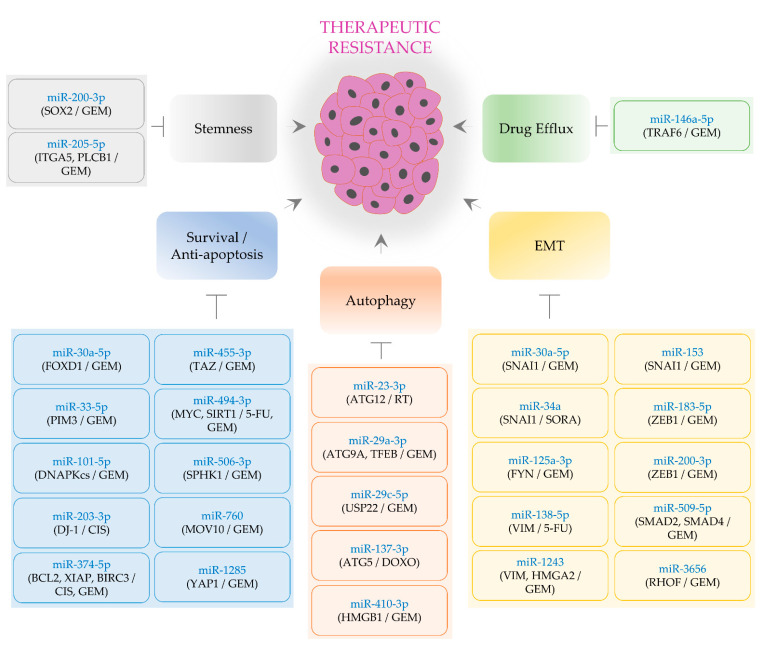
MiRNA-mediated repression of therapeutic resistance in PaC. Tumor-suppressive miRNAs in rounded rectangles are shown in blue. Round brackets denote target genes of miRNAs and then therapeutic agents affected by miRNAs. Activation is indicated by an arrow. Inhibition is denoted by a perpendicular line. GEM: gemcitabine; 5-FU: 5-fluorouracil; PTX: paclitaxel; CIS: cisplatin; RT: radiotherapy; DOXO: doxorubicin; SORA: sorafenib.

Tumor-suppressive miR-30a-5p was observed to restrain EMT process and metastasis [100,101,102]. A deep sequencing analysis of small RNAs revealed that miR-30a-5p is one of the negatively regulated miRNAs in gemcitabine-resistant PaC cells [103]. It was also indicated that this miRNA targets SNAI1 and that the growth of PaC is synergistically suppressed by the co-treatment with miR-30a-5p and gemcitabine in vivo. These results implicate that the miR-30a-5p/SNAI1 axis is a feasible therapeutic choice for PaC [103] (Figure 2 and Table 2).

#### 3.1.2. MiR-34a

The progression of PaC is impeded by miR-34a, which targets SNAI1 [139]. In another study, miR-34a was found to improve the anti-cancer efficacy of sorafenib [110] (Figure 2 and Table 2). Especially, the overexpression of miR-34a augments the sorafenib-mediated inhibition of the intrahepatic growth of PaC in vivo [110]. Besides, it was demonstrated that the expression of miR-34a is repressed by DNA methyltransferase-mediated hypermethylation of the miR-34a promoter. The knockdown of DNA methyltransferase restores miR-34a levels and downregulates EMT markers, such as SNAI1 and TWIST [110], suggesting that the anti-cancer effects of DNA methyltransferase inhibition are at least partly through transcriptionally activating miR-34a expression.

#### 3.1.3. MiR-125a-3p

Although miR-125a-5p is an EMT-promoting factor (see Section 2.1.2), the EMT process can be subdued by miR-125a-3p that is generated from the same miRNA precursor. It was indicated that the effect of gemcitabine is increased by miR-125a-3p, which represses EMT by targeting proto-oncogene C-Fyn (*FYN*) [113] (Figure 2 and Table 2). The expression of miR-125a-3p and miR-125a-5p is downregulated and upregulated, respectively, in PaC tissues [140,141]. These findings suggest that the differential stability of miR-125a-3p and miR-125a-5p is regulated by undiscovered specific degradation factors, contributing to therapeutic resistance.

#### 3.1.4. MiR-138-5p and MiR-153

It has been noted that miR-138-5p performs a tumor-suppressive function by regulating migration, invasion, and EMT in breast and colorectal cancer [142,143]. Furthermore, miR-153 is recognized to suppress EMT and metastasis in oral cancer, breast cancer, as well as hepatocellular carcinoma [144,145]. In addition, both miR-138-5p and miR-153 have been proposed to inhibit the progression of PaC through regulating proliferation, migration, and invasion [146,147]. Moreover, it was validated that miR-138-5p targets vimentin (*VIM*) and increases the anti-proliferative effect of 5-FU in vitro. Moreover, miR-153, which targets SNAI1, reinforces the inhibitory effects of gemcitabine on cell viability in vitro and the growth of PaC cells in vivo [116,120] (Figure 2 and Table 2). These findings demonstrate the role of them as bona fide EMT- and therapeutic resistance-suppressing miRNAs.

#### 3.1.5. MiR-183-5p and MiR-200-3p

Kruppel-like factor 4 (*KLF4*) has been considered as a tumor-suppressive transcription factor in PaC [148,149]. KLF4 overexpression significantly decreases cell proliferation via inducing cyclin-dependent kinase inhibitor 1A (*CDKN1A*, also called *p21CIP1*) expression [148]. Furthermore, KLF4 can subdue EMT and metastasis by downregulating caveolin-1 levels [149]. Further, it was recently exhibited that gemcitabine treatments result in an increase in ZEB1 levels, together with a reduction of KLF4, miR-183-5p, and miR-200-3p [18]. The knockdown of KLF4 upregulates ZEB1 via restraining the level of miR-183-5p and miR-200-3p, both of which directly target ZEB1. In addition, gemcitabine resistance is attenuated by the overexpression of either KLF4, miR-183-5p, or miR-200-3p. Monitoring of in vivo PaC growth revealed that KLF4 overexpression enhances the efficacy of gemcitabine [18] (Figure 2 and Table 2).

#### 3.1.6. MiR-509-5p and MiR-1243

Screening assays using a cell-based reporter system identified miR-509-5p and miR-1243 as EMT-inhibiting factors [133]. Target validation experiments showed that miR-509-5p directly interacts with the 3′ UTR of SMAD family member 2 (*SMAD2*) and SMAD4. Moreover, miR-1243 was determined to target VIM and high mobility group AT-hook 2 (*HMGA2*). Besides, the effectiveness of gemcitabine is improved in miR-509-5p- or miR-1243-overexpressing PaC cells [133] (Figure 2 and Table 2).

#### 3.1.7. MiR-3656

Ras homolog family member F (*RHOF*) exerts oncogenic effects through promoting EMT and metastasis [150]. In PaC, RHOF knockdown leads to an increase in EMT-promoting factors, such as VIM and TWIST1 [138]. RHOF is targeted by miR-3656, and the cytotoxicity of gemcitabine is ameliorated in miR-3656-overexpressing cells. Further, TWIST1 overexpression interferes with the chemosensitization effect of miR-3656 in vitro. It was also confirmed that miR-3656 enhances gemcitabine-induced growth inhibition, along with a decrease in VIM and TWIST1 levels in vivo [138] (Figure 2 and Table 2). These observations suggest that the miR-3656/RHOF/EMT axis notably modulates the responsiveness of cancer cells to gemcitabine.

### 3.2. Stemness-Regulating MiRNAs

#### 3.2.1. MiR-200-3p

Several studies have proved that miR-200-3p distinctly inhibits cancer stemness [151,152,153,154,155]. In particular, miR-200-3p inhibits the self-renewal of CSCs via targeting SRY-box transcription factor 2 (*SOX2*), a stemness gene [155]. Another study also showed that miR-200-3p can indirectly modulate the expression of CD44, a CSC maintenance factor, via targeting fascin-1 (*FSCN1*) [156]. In PaC, the low expression of miR-200-3p was observed in CSCs. Both colony formation ability of CSCs and gemcitabine resistance are attenuated by miR-200-3p overexpression [122] (Figure 2 and Table 2). These results indicate that miR-200-3p restores gemcitabine sensitivity by modulating EMT and stemness (also see Section 3.1.5).

#### 3.2.2. MiR-205-5p

Growing evidence has revealed that miR-205-5p acts as a stemness-attenuating miRNA by inhibiting several genes, including integrin subunit alpha 5 (*ITGA5*) and phospholipase C beta 1 (*PLCB1*) [157,158,159]. In PaC, miR-205-5p overexpression brings about the reduction of CSC populations in gemcitabine-resistant cells in vitro. Further, an experimental observation demonstrated that miR-205-5p overexpression makes gemcitabine more effective in inhibiting the growth of resistant cells in vivo [124] (Figure 2 and Table 2).

### 3.3. Cell Survival- and Apoptosis-Regulating MiRNAs

#### 3.3.1. MiR-30a-5p

As mentioned in Section 3.1.1, miR-30a-5p can modulate the effect of gemcitabine on cancer cells. In addition to this, miR-30a-5p is able to target forkhead box D1 (*FOXD1*), an upstream activator of ERK signaling. As a consequence, the overexpression of miR-30a-5p can induce apoptosis in vitro and potentiate the anti-cancer activity of gemcitabine in vivo [108] (Figure 2 and Table 2).

#### 3.3.2. MiR-33-5p, MiR-101-5p, MiR-203-3p, and MiR-506-3p

AKT inhibits the expression and activity of pro-apoptotic factors, such as BAD and caspase-9, consequently impairing the apoptotic cascade and contributing to gemcitabine resistance [160,161,162]. Moreover, since AKT can be activated by gemcitabine exposure [163], targeting of AKT is promising to advance gemcitabine efficacy. Several studies have found that miR-33-5p, miR-101-5p, and miR-506-3p sensitize cells to gemcitabine and that miR-203-3p reverses cisplatin resistance in PaC [109,112,123,132]. All these miRNAs have in common that they impose a constraint on AKT activation. Specifically, miR-33-5p and miR-101-5p negatively regulate AKT activation via targeting serine/threonine-protein kinase Pim-3 (*PIM3*) and DNA-dependent protein kinase catalytic subunit (*DNA-PKcs*), respectively. Further, miR-203-3p and miR-506-3p straightly target protein/nucleic acid deglycase DJ-1 (*DJ-1*) and sphingosine kinase 1 (*SPHK1*), respectively (Figure 2 and Table 2).

#### 3.3.3. MiR-374-5p

In breast cancer, miR-374-5p promotes cell proliferation, survival, migration, and invasion [164]. By contrast, miR-374-5p performs a tumor-suppressive function in lung and bladder cancer and is associated with overall patient survival [165,166]. In PaC, miR-374-5p attenuates therapeutic resistance. PaC cells transfected with miR-374-5p exhibit a high degree of apoptosis following treatments with gemcitabine in vitro [127]. In this study, it was noticed that miR-374-5p potentiates gemcitabine efficacy, thereby extending survival in a xenograft mouse model of PaC. Moreover, the effect of cisplatin tends to be increased by miR-374-5p in resistant cells [128]. Such resistance-alleviating effects of miR-374-5p can be due to the direct inhibition of several anti-apoptotic genes, such as BCL2, X-linked inhibitor of apoptosis (*XIAP*), and baculoviral IAP repeat-containing 3 (*BIRC3*) [127] (Figure 2 and Table 2).

#### 3.3.4. MiR-455-3p and MiR-1285

Tafazzin (*TAZ*), a YAP homolog, is responsible for therapeutic resistance and is inactivated by the Hippo pathway. The blocking of YAP/TAZ signaling is expected to reduce the development of therapeutic resistance [167,168]. Gemcitabine efficacy can be augmented by atorvastatin, which suppresses YAP/TAZ signaling [169] (also see Section 2.3.4 describing the Hippo pathway and YAP1). In PaC, the downregulation of miR-455-3p and miR-1285 aggravates gemcitabine resistance. On the other hand, the overexpression of these miRNAs leads to the improvement of gemcitabine efficacy [130,137]. In their study, it was confirmed that TAZ and YAP1 are directly modulated by miR-455-3p and miR-1285, respectively (Figure 2 and Table 2).

#### 3.3.5. MiR-494-3p

Both proto-oncogene c-Myc (*MYC*) and sirtuin 1 (*SIRT1*) are highly expressed in PaC [170,171]. The silencing of either MYC or SIRT1 can stimulate apoptosis induction, thus increasing the anti-cancer activity of several agents, such as 5-FU and gemcitabine [172,173]. Further, it was shown that both MYC and SIRT1 can be targeted by miR-494-3p. Accordingly, PaC cells are sensitized to 5-FU and gemcitabine by miR-494-3p restoration [131] (Figure 2 and Table 2). It is noteworthy that the metastasis of hepatocellular carcinoma is accelerated by miR-494-3p [174], indicating that miR-494-3p plays context-specific functions.

#### 3.3.6. MiR-760

Generally, integrins mediate cell survival signaling by activating focal adhesion kinase (*FAK*) [175]. Further, it was indicated that integrin subunit beta 1 (*ITGB1*) can facilitate metastasis and confer therapeutic resistance in PaC [176,177]. In addition, a recent study denoted that ITGB1 is post-transcriptionally stabilized by Mov10 RISC complex RNA helicase (*MOV10*) and that miR-760 destabilizes ITGB1 by targeting MOV10. Owing to this ability, miR-760 can elevate gemcitabine efficacy in PaC [136] (Figure 2 and Table 2). Moreover, since MOV10 facilitates angiogenesis [178], miR-760 may serve as an angiogenesis and metastasis suppressor via the MOV10/ITGB1 axis.

### 3.4. Autophagy-Inhibiting MiRNAs

#### 3.4.1. MiR-23-3p and MiR-137-3p

Lipidation of LC3I to LC3II is necessary for autophagosome formation and is known to be facilitated by ATG5 and ATG12 [179]. In connection with therapeutic resistance, the inhibition of either ATG5 or ATG12 can sensitize cells to therapeutic agents [180,181]. Further, it was reported that the effectiveness of radiotherapy and doxorubicin is advanced by miR-23-3p and miR-137-3p, respectively [105,114]. These miRNAs inhibit overall cell viability in vitro and enhance the ability of anti-cancer therapies to impede the in vivo growth of PaC. Such improvement of therapeutic responses is due to the fact that ATG12 and ATG5 are targeted by miR-23-3p and miR-137-3p, respectively [105,114] (Figure 2 and Table 2).

#### 3.4.2. MiR-29a-3p

ATG9A functions as one of the essential components for the autophagy process by controlling the generation of phosphatidylinositol-4-phosphate, which promotes autophagosome–lysosome fusions [182]. In addition, transcription factor EB (*TFEB*) induces autophagy via regulating the level of autophagy and lysosomal genes [183]. Both ATG9A and TFEB facilitate the process of autophagy, and they were validated as miR-29a-3p target genes in PaC. Furthermore, the sensitivity of cells to gemcitabine is increased by miR-29a-3p [106] (Figure 2 and Table 2).

#### 3.4.3. MiR-29c-5p

Ubiquitin-specific-processing protease 22 (*USP22*) has been recognized to promote EMT process and metastasis via activating FAK and repressing anti-cancer immunity in PaC [184,185]. USP22 also increases LC3II and autophagosome levels so that USP22 can enhance gemcitabine resistance through activating autophagy [186]. Moreover, it was revealed that miR-29c-5p increases the cytotoxic potency of gemcitabine through inhibiting USP22-mediated autophagy in vitro. In a xenograft mouse model, the overexpression of miR-29c-5p also suppresses autophagy, sensitizing PaC cells to gemcitabine [107] (Figure 2 and Table 2).

#### 3.4.4. MiR-410-3p

High-mobility group box 1 (*HMGB1*) is capable of promoting autophagy by disengaging BCL2 from beclin-1, an autophagy factor [187]. In PaC, it was confirmed that HMGB1 promotes metastasis and that its expression is escalated in gemcitabine-resistant cells [188,189]. Furthermore, a recent study denoted that miR-410-3p targets HMGB1 to exert negative effects on gemcitabine resistance in PaC [129] (Figure 2 and Table 2).

### 3.5. MiRNAs Regulating Drug Efflux

#### MiR-146a-5p

In addition to the regulation of ABCB1 expression by Wnt/β-catenin signaling (Section 2.4), NF-κB positively controls the level of ABCB1, hence prompting therapeutic resistance [190,191]. Recently, it was revealed that miR-146a-5p can sensitize PaC cells to gemcitabine [119]. The overexpression of miR-146a-5p enhances the cytotoxicity of gemcitabine by increasing apoptosis rates in vitro and in vivo. Mechanistically, miR-146a-5p targets TNF receptor-associated factor 6 (*TRAF6*) to downregulate ABCB1 levels via inactivating NF-κB signaling [119] (Figure 2 and Table 2).

## 4. CircRNA, lncRNA, and Therapeutic Resistance

### 4.1. LncRNAs Alleviating Therapeutic Resistance

#### 4.1.1. LncRNA-AB209630

**Figure 3:**
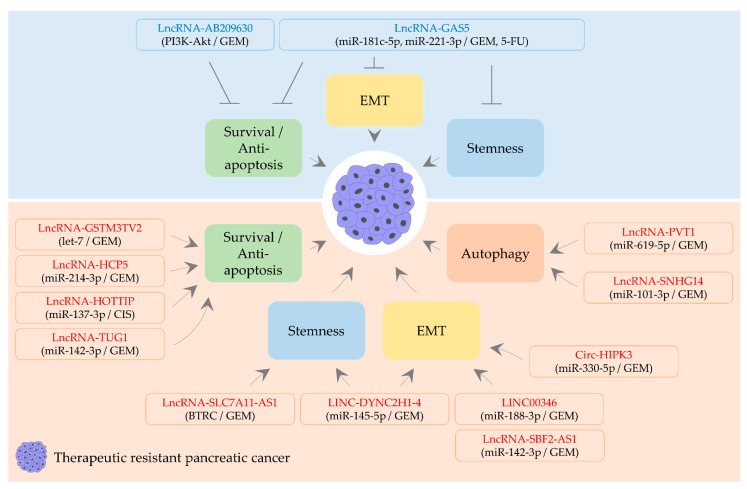
CircRNA- and lncRNA-mediated regulation of therapeutic resistance in PaC. Tumor-suppressive lncRNAs in rounded rectangles are shown in blue. Oncogenic circRNA and lncRNA are indicated in red within rounded rectangles. Round brackets denote miRNAs, the signaling pathway, or a protein molecule affected by ncRNAs and then therapeutic agents influenced by ncRNAs. Activation is indicated by an arrow. Inhibition is denoted by a perpendicular line. GEM: gemcitabine; 5-FU: 5-fluorouracil; CIS: cisplatin.

Although it is necessary to uncover the precise mechanism, it has been reported that lncRNA-AB209630 can perform tumor-suppressive functions. In hepatocellular carcinoma, the level of lncRNA-AB209630 is low, and the overexpression of lncRNA-AB209630 restrains the migration and invasion of cells [192]. Moreover, lncRNA-AB209630 significantly induces apoptotic cell death and inhibits cell proliferation, as well as invasion in hypopharyngeal cancer [193]. In this study, it was also noticed that the low expression of lncRNA-AB209630 is correlated with poor prognosis. Furthermore, it was observed that lncRNA-AB209630 suppresses proliferation, colony formation, and PI3K/AKT activities in gemcitabine-resistant PaC cells [194]. These results suggest that lncRNA-AB209630 can reverse gemcitabine resistance, at least partly via modulating pro-survival signaling (Figure 3 and Table 3).

#### 4.1.2. LncRNA-GAS5

The level of lncRNA-GAS5 is reduced in many cancer types, and this lncRNA negatively regulates cell survival, proliferation, migration, and EMT [199]. LncRNA-GAS5 was noticed to inactivate miR-32-5p and suppress metastasis by upregulating PTEN levels [200]. Moreover, lncRNA-GAS5 can serve as a sponge for miR-181c-5p [46] (see Section 2.3.4 and Table 1 about miR-181c-5p). Such roles of lncRNA-GAS5 as competitive endogenous RNAs affects the resistance status of cancer. The silencing of lncRNA-GAS5 desensitizes PaC cells to both 5-FU and gemcitabine by inactivating Hippo signaling [46] (Figure 3 and Table 3).

Furthermore, lncRNA-GAS5 antagonizes miR-221-3p [48], which promotes therapeutic resistance by promoting EMT and stemness (see Section 2.1.3 and Section 2.2.1, and Table 1 about miR-221-3p). The overexpression of lncRNA-GAS5 inhibits EMT and stemness, thus reversing gemcitabine resistance in vitro. Moreover, in vivo experiments demonstrated that lncRNA-GAS5 restrains metastasis and reinforces the growth inhibitory effect of gemcitabine [48]. Additionally, it was remarked that miR-221-3p targets suppressor of cytokine signaling 3 (*SOCS3*) [48] (Figure 3 and Table 3). SOCS3 is a negative regulator of Janus kinase/STAT3 signaling, which facilitates metastasis, EMT, and stemness [201].

### 4.2. A circRNA and LncRNAs Aggravating Therapeutic Resistance

#### 4.2.1. Circ-HIPK3

Circ-HIPK3 is one of the upregulated circRNAs and positively regulates cell growth, survival, and metastasis in colorectal and renal cancer [202,203]. However, this circRNA can impede metastasis in bladder cancer [204], indicating its double-edged role. In PaC, circ-HIPK3 worsens gemcitabine resistance via hampering miR-330-5p, an EMT-inhibiting miRNA (Figure 3, Table 2 and Table 3). The depletion of circ-HIPK3 reduces cell proliferation, migration, invasion, and EMT of gemcitabine-resistant cells [126], implying that the circ-HIPK3/miR-330-5p/EMT axis may regulate the effect of other cancer therapies.

#### 4.2.2. LINC00346

LINC00346 plays a critical role in several aspects of cancer progression. LINC00346 is responsible for glioma angiogenesis by stimulating the migration and tube formation of glioma-associated endothelial cells [205]. Furthermore, LINC00346 is upregulated in colorectal cancer tissues, inhibits apoptotic cell death, and triggers cell proliferation, migration, as well as invasion [206]. In addition, LINC00346 promotes cisplatin resistance in nasopharyngeal cancer partly via sponging miR-342-5p, a tumor-suppressive miRNA [207]. In PaC, the depletion of LINC00346 renders cells susceptible to gemcitabine by increasing the level of miR-188-3p and caspase-3 activities in vitro. The inhibitory effect of gemcitabine on PaC growth is augmented by LINC00346 silencing in xenografts [121] (Figure 3, Table 2 and Table 3). In support of this finding, it was observed that miR-188-3p exerts a gemcitabine-sensitizing activity by targeting bromodomain-containing 4 (*BRD4*) [121], which can facilitate NF-κB-dependent EMT [208].

#### 4.2.3. LINC-DYNC2H1-4

It has been suggested that miR-145-5p negatively affects EMT and stemness, for example, by suppressing NF-κB signaling and targeting SRY-box transcription factor 9 (*SOX9*) [209,210]. Interestingly, both EMT and stemness of gemcitabine-resistant PaC cells are attenuated by the knockdown of LINC-DYNC2H1-4, which inhibits miR-145-5p activities [118] (Figure 3, Table 2 and Table 3). Mechanically, it was further shown that miR-145-5p targets numerous genes involved in the regulation of EMT and stemness, namely, ZEB1, SOX2, lin-28 homolog (*LIN28*), nanog homeobox (*NANOG*), and POU class 5 homeobox 1 (*POU5F1*, also called *OCT4*) [118].

#### 4.2.4. LncRNA-GSTM3TV2

The overexpression of lncRNA-GSTM3TV2 abates apoptosis induced by gemcitabine in vitro. Moreover, this lncRNA diminishes in vivo efficacy of gemcitabine, as evidenced by the measurement of PaC growth [104]. One of the validated mechanisms whereby lncRNA-GSTM3TV2 promotes gemcitabine resistance includes the lncRNA-mediated downregulation of let-7 (Figure 3, Table 2 and Table 3). Besides, let-7 was confirmed to target linker for activation of T-cells family member 2 (*LAT2*) and oxidized low-density lipoprotein receptor 1 (*OLR1*) [104]. LAT2, a transporter of neutral amino acids, activates mechanistic target of rapamycin kinase (mTOR), thereby inhibiting apoptotic cell death [211]. OLR1 is also known to impair apoptosis via activating NF-κB [212].

#### 4.2.5. LncRNA-HCP5 and lncRNA-HOTTIP

Therapeutic resistance is also modulated by lncRNA-HCP5 and lncRNA-HOTTIP, both of which exhibit anti-apoptotic functions in PaC. The silencing of either lncRNA-HCP5 or lncRNA-HOTTIP triggers in vitro apoptosis following treatments with gemcitabine or cisplatin, respectively [115,125]. Their effects on therapeutic agents can be due to the abolishment of miRNA activities. LncRNA-HCP5 interrupts miR-214-3p activities, augmenting the level of heparin-binding growth factor (*HDGF*) [125]. Furthermore, lncRNA-HOTTIP interacts with and inactivates miR-137-3p [115] (Figure 3, Table 2 and Table 3). In terms of apoptosis, miR-137-3p overexpression can induce cell death via attenuating XIAP levels [213].

#### 4.2.6. LncRNA-PVT1

Therapeutic resistance can be promoted by an autophagy-promoting lncRNA. Through sponging miR-619-5p, lncRNA-PVT1 upregulates the expression of ATG14 and promotes autophagic activity [135]. LncRNA-PVT1 suppresses gemcitabine-induced caspase activations and apoptotic cell death in vitro. Further, the suppressive effect of gemcitabine on the growth of PaC is weakened by lncRNA-PVT1 in vivo [135] (Figure 3, Table 2 and Table 3). LncRNA-PVT1 also enhances cell proliferation and EMT [196,214], indicating that lncRNA-PVT1 is a bona fide oncogenic factor in PaC.

#### 4.2.7. LncRNA-SBF2-AS1

Twinfilin actin-binding protein 1 (*TWF1*) has been noticed to provoke EMT and chemoresistance. For example, TWF1-silencing breast cancer cells undergo a mesenchymal-to-epithelial transition. Moreover, the cytotoxicity of doxorubicin and paclitaxel is enhanced by TWF1 knockdown [215]. In PaC, lncRNA-SBF2-AS1 can interfere with miR-142-3p activities, resulting in an increase in TWF1 levels and gemcitabine resistance [117]. The depletion of lncRNA-SBF2-AS1 was observed to increase apoptotic cell death and suppress EMT in gemcitabine-resistant cells [117] (Figure 3, Table 2 and Table 3). Consistent with these findings, it has been indicated that miR-142-3p functions as a metastasis and EMT repressor [216,217].

#### 4.2.8. LncRNA-SLC7A11-AS1

Nuclear factor erythroid 2-related factor 2 (*NFE2L2*, also called *NRF2*) has antioxidant properties through transcriptionally stimulating the expression of antioxidant genes such as glutathione S-transferases [218]. The expression of NFE2L2 is controlled by proteasomal degradation via the SKP1-CUL1-F-box protein (SCF) complex [219]. In addition, cancer stemness is known to be suppressed by beta-transducin repeat containing E3 ubiquitin-protein ligase (*BTRC*, also known as *β-TrCP*), one of the SCF components [220]. Recently, it was ascertained that lncRNA-SLC7A11-AS1 promotes cancer stemness via scavenging reactive oxygen species (ROS) and that the silencing of lncRNA-SLC7A11-AS1 re-sensitizes resistant cells to gemcitabine [197]. The knockdown of lncRNA-SLC7A11-AS1 strengthens the suppressive effect of gemcitabine on colony formation in vitro and the growth of PaC in vivo (Figure 3 and Table 3). Mechanically, it was proven that lncRNA-SLC7A11-AS1 binds to BTRC proteins and prevents BTRC-mediated degradation of NFE2L2 [197].

#### 4.2.9. LncRNA-SNHG14

In addition, lncRNA-SNHG14 contributes to gemcitabine resistance via activating autophagy in PaC [111]. This study showed that miR-101-3p interacts with lncRNA-SNHG14 and reverses lncRNA-SNHG14-mediated gemcitabine resistance by attenuating autophagy-related factors, ATG4D and RAS-associated protein RAB5A (*RAB5A*) (Figure 3, Table 2 and Table 3).

#### 4.2.10. LncRNA-TUG1

In a similar vein, lncRNA-TUG1 can activate ERK and desensitize PaC cells to gemcitabine. The depletion of lncRNA-TUG1 induces apoptotic cell death and enhances the cytotoxicity of gemcitabine [198]. Recent studies demonstrated that lncRNA-TUG1 exacerbates cisplatin resistance in bladder cancer. Furthermore, lncRNA-TUG1 inactivates miR-142-3p, thereby hastening metastasis and EMT in hepatocellular carcinoma [221]. Regarding miR-142-3p, it was reported that this miRNA induces apoptotic cell death by targeting heat shock 70 KDa protein 1B (*HSPA1B*) in PaC [222]. These observations suggest a possibility that lncRNA-TUG1 regulates the susceptibility of cells to gemcitabine by blocking the activity of miR-142-3p in PaC (Figure 3, Table 2 and Table 3) (also see Section 4.2.7 about miR-142-3p).

## 5. Conclusions

Efforts have been made to discover possible and efficacious combination strategies for subjugating therapeutic resistance, a prevalent and severe problem for curing cancer. In addition, it has been suggested that combination therapy using mechanistically diverse agents is beneficial for cancer treatment [223,224,225,226]. For example, ERK inhibition induces the compensatory activation of PI3K/AKT, and simultaneous PI3K inhibition synergistically augments the anti-cancer efficacy of an ERK inhibitor [226]. In this respect, targeting ncRNAs is fascinating since a single ncRNA is capable of controlling multiple signaling pathways in cells. Moreover, ncRNAs can regulate the cancer microenvironment, contributing to disease progression and therapeutic resistance [227]. A ncRNA-based therapy through the depletion or restoration of ncRNAs has been perceived to strikingly boost the effects of anti-cancer treatments in cancer [228,229]. Moreover, experimental evidence presented here demonstrated that ncRNA-based therapy is a potential strategy to surmount the therapeutic resistance of currently available treatments, such as chemotherapy and radiation therapy, in PaC.

A recent investigation indicated that miRNAs selectively advance the efficacy of drugs. For example, miR-326 strengthens the anti-cancer effect of gefitinib but not that of doxorubicin. Moreover, the effect of a miRNA on drug efficacy is different between breast cancer cell lines [230]. In addition, the application of miRNA primary/precursor forms for cancer treatments requires a concern about the opposite function of miR-3p and -5p (Section 3.1.3). Therefore, more investigations on the function of miRNAs and the relationship between miRNAs and anti-cancer agents are warranted to find highly effective combination pairs. Further, even though lncRNA-SNHG14 acts as a gemcitabine resistance factor in PaC (Section 4.2.9), lncRNA-SNHG14 is able to suppress invasion and promote apoptotic cell death via sponging miR-92a-3p in glioblastoma [231], showing its dual role in cancer (also see Section 4.2.1 about the dual role of circ-HIPK3). Regarding miR-92a-3p, it was reported that this miRNA serves as an oncogenic miRNA by accelerating cell proliferation and metastasis in PaC [232]. Additionally, circ-HIPK3 and lncRNA-TUG1 can interact with miR-421 and miR-197-3p, respectively [233,234], and both miRNAs are also ascertained as oncogenic factors in PaC [235,236]. These findings demonstrate a possibility of the sequestration of oncogenic miRNAs in other oncogenic ncRNAs. Are some oncogenic miRNAs reactivated, contributing to compensatory activation of signaling pathways in oncogenic ncRNA-depleted cells? More experimental and bioinformatic approaches for comprehensive analyses of circRNA/lncRNA-miRNA networks are necessary. Ongoing endeavors to understand the detailed feature of ncRNAs will provide unique opportunities to invent better ncRNA-based therapeutic strategies for PaC.

## Figures and Tables

**Table 1:** Oncogenic miRNAs that reinforce therapeutic resistance in PaC.

miRNA	Expression	In Vivo Experiment and/or Clinical Relevance	Ref.
miR-10a-5p	Increased in gemcitabine-resistant AsPC-1 cells. Upregulated in cancer tissues compared to matched adjacent tissues	Subcutaneous injections of AsPC-1 cells transduced with miR-10a-5p lentiviral plasmids. A positive correlation with unfavorable prognosis of patients	[37]
miR-17-5p	Augmented in MIAPaCa-2 cells overexpressing GFRA2. Escalated in cancer tissues	Positively correlated with poor survival	[39]
miR-21-5p	Upregulated in stem-like cells isolated from gemcitabine-resistant L3.6pl cells (GR-L3.6pl)	Orthotopic injections of stem-like cells from GR-L3.6pl following miR-21-5p knockdown	[40]
	Upregulated in 5-FU-resistant PATU8988 cells	-	[41]
miR-29-3p	Highly expressed in MIAPaCa-2, PSN-1, and PANC-1 cells compared to BxPC-3 cells	-	[42]
miR-125a-5p	Upregulated in cancer tissues from chemo-resistant patients	Inversely correlated with the level of a target gene (*TNFAIP3*)	[43]
miR-135-5p	High expression in cancer tissues compared to normal controls	Subcutaneous injections of miR-135-5p-overexpressing MIAPaCa-2 cells or miR-135-5p knockdown PANC-1 cells followed by gemcitabine treatment. Short overall survival of patients with high miR-135-5p levels	[44]
miR-181c-5p	High expression in cancer tissues compared to normal controls. Upregulated in gemcitabine-resistant SW1990 cells and 5-FU-resistant PATU8988 cells	Poor overall survival of patients with strong miR-181c-5p expression	[45,46]
miR-221-3p	Upregulated in 5-FU-resistant PATU8988 cells	Negative correlation with the overall survival of patients	[47]
	Upregulated in stem-like cells isolated from gemcitabine-resistant L3.6pl cells (GR-L3.6pl)	Orthotopic injections of stem-like cells from GR-L3.6pl following miR-221-3p knockdown	[40]
	Upregulated in cancer tissues compared to normal controls	-	[48]
miR-223-3p	Highly abundant in gemcitabine-resistant AsPC-1 and PANC-1 cells	-	[49]
	Downregulated by genistein in gemcitabine-resistant AsPC-1 and BxPC-3 cells	Subcutaneous injections of gemcitabine-resistant BxPC-3 cells + intratumor injection of miR-223-3p inhibitors or genistein (15 mg/kg, oral administration)	[50]
	Upregulated in cisplatin-resistant BxPC-3 cells	-	[51]
miR-296-5p	High expression in MIAPaCa-2, PK-8, and PK-45H cells	Negative correlation with the overall survival of patients	[52]
miR-301-3p	Upregulated in CFPAC-1 and BxPC-3 cells under hypoxia	Aggressive cancer behaviors and poor overall survival in patients with elevated miR-301-3p expression	[53,54]
	Heightened in gemcitabine-resistant SW1990 and PANC-1 cells (GR-PANC-1)	Intraperitoneal injections of miR-301-3p inhibitors and gemcitabine (20 mg/kg) into mice bearing GR-PANC-1 xenografts	[55]
miR-320a	Upregulated in 5-FU-resistant PATU8988 cells	-	[56]
miR-331-3p	Upregulated in gemcitabine-resistant PANC-1 and MIAPaCa-2 cells. Increased in plasma from patients receiving chemotherapy	-	[57]
miR-342-3p	Highly expressed in gemcitabine-resistant cancer tissues from patients	Intraperitoneal injections of gemcitabine (12.5 mg/kg) into orthotopic xenograft mouse models established using miR-342-3p-overexpressing MIAPaCa-2 cells	[58]
miR-1246	Highly abundant in gemcitabine-resistant PANC-1 cells	Negatively correlated with the overall survival of patients	[59]
miR-1266-5p	Upregulated in cancer tissues compared to normal controls	Tail vein injections of miR-1266-5p inhibitors into mice bearing AsPC-1 xenograft + intraperitoneal injection of gemcitabine (50 mg/kg). Positively correlated with unfavorable prognosis of patients	[60]

**Table 2:** Tumor-suppressive miRNAs that abate therapeutic resistance in PaC.

miRNA	Expression	In Vivo Experiment and/or Clinical Relevance	Ref.
let-7	Negatively regulated by lncRNA-GSTM3TV2	-	[104]
miR-23-3p	Reduced in radioresistant PANC-1 and BxPC-3 cells	Radiotherapy (10-Gy) following the establishment of xenograft mouse models using miR-23-3p-overexpressing cells	[105]
miR-29a-3p	Low expression in PANC-1, BxPC-3, MIAPaCa-2, and COLO357 cells compared to normal pancreatic ductal epithelial cells	-	[106]
miR-29c-5p	-	Intraperitoneal injections of gemcitabine (50 mg/kg) into mice bearing miR-29c-5p-overexpressing PANC-1 cells	[107]
miR-30a-5p	Downregulated in gemcitabine-resistant SW1990 cells	Subcutaneous injections of miR-30a-5p-overexpressing SW1990 cells followed by gemcitabine treatment (50 mg/kg)	[103]
	Low expression in cancer cell lines (PANC-1, MIAPaCa-2, and BxPC-3) and cancer tissues	Injections of miR-30a-5p into BxPC-3 xenografts followed by gemcitabine administration (100 mg/kg). Poor overall survival of patients with low miR-30a-5p expression	[108]
miR-33-5p	Lowered in plasma and cancer tissues from patients	Poor overall survival of patients with low miR-33-5p expression	[109]
miR-34a	Promoter is highly methylated in cancer tissues compared to paired normal tissues	Oral administration of sorafenib (1.0 mg/kg) in mice bearing xenografts of miR-34a-overexpressing PANC-1 cells	[110]
miR-101-3p	Downregulated in cancer tissues	-	[111]
miR-101-5p	Lowered in gemcitabine-resistant cancer tissues	-	[112]
miR-125a-3p	Reduced in gemcitabine-treated PATU8988 and PANC-1 cells	-	[113]
miR-137-3p	Decreased by doxorubicin treatments in PANC-1 cells. Low expression in cancer cell lines (PANC-1, HS766T, AsPC-1)	Intravenous injections of doxorubicin (5 mg/kg) in mice bearing xenografts of miR-137-overexpressing PANC-1 cells	[114,115]
miR-138-5p	Downregulated in primary cancer tissues compared to normal controls	-	[116]
miR-142-3p	Lowered in gemcitabine-resistant PANC-1 and AsPC-1 cells	-	[117]
miR-145-5p	Downregulated in gemcitabine-resistant BxPC-3 cells	-	[118]
miR-146a-5p	Decreased in cancer tissues compared to adjacent normal tissues	Intra-tumoral injections of miR-146a-5p + intraperitoneal injection of gemcitabine (20 mg/kg). Short overall survival of patients with low miR-146a-5p expression	[119]
miR-153	Downregulated in cancer tissues compared to normal tissues. Low expression in gemcitabine-resistant PANC-1, Capan-2, and AsPC-1 cells	Intraperitoneal injections of gemcitabine (50 mg/kg) in mice bearing xenografts of miR-153-overexpressing AsPC-1 cells. Unfavorable overall survival of patients with low miR-153 expression	[120]
miR-183-5p	Reduced in PANC-1 and BxPC-3 cells following gemcitabine exposure	Intraperitoneal injections of gemcitabine (80 mg/kg) in mice bearing xenografts of KLF4-overexpressing PANC-1 cells	[18]
miR-188-3p	-	Poor overall survival of patients with low miR-188-3p expression	[121]
miR-200-3p	Reduced in PANC-1 and BxPC-3 cells following gemcitabine exposure	Intraperitoneal injections of gemcitabine (80 mg/kg) in mice bearing xenografts of KLF4-overexpressing PANC-1 cells	[18]
	Low expression in CD24^+^/CD44^+^/epithelial-specific antigen (ESA)^+^ CSCs	-	[122]
miR-203-3p	Downregulated in cisplatin-resistant SW1990 cells	-	[123]
miR-205-5p	Decreased in primary cancer lesions	Intravenous injections of gemcitabine-conjugated micelles into mice bearing xenografts of miR-205-5p-overexpressing MIAPaCa-2 cells	[124]
miR-214-3p	Downregulated in gemcitabine-resistant cancer tissues	-	[125]
miR-330-5p	Reduced in cancer tissues compared to tissues of normal pancreas	-	[126]
miR-374-5p	Repressed in cancer tissues compared to adjacent normal tissues	Intraperitoneal injections of gemcitabine (50 mg/kg) into xenograft mouse models established using miR-374-5p-overexpressing AsPC-1 cells	[127]
	Downregulated in cisplatin-resistant BxPC-3 cells	-	[128]
miR-410-3p	Downregulated in human cancer xenografts from gemcitabine-treated mice	Low miR-410-3p expression is correlated with short overall survival of patients	[129]
miR-455-3p	Decreased in cell lines (PANC-1 and MIAPaCa-2 cells) and cancer tissues	-	[130]
miR-494-3p	Downregulated in cancer tissues compared to tissues of normal pancreas	Low miR-494-3p expression is correlated with distant metastasis and poor overall survival of patients	[131]
miR-506-3p	Low expression in cancer tissues compared to normal controls	Short overall survival of patients with low miR-506-3p expression	[132]
miR-509-5p	Downregulated in cancer tissues compared to noncancerous adjacent tissues	Worse overall survival of patients with low miR-509-5p levels	[133,134]
miR-619-5p	Reduced in gemcitabine-treated PANC-1 cells	-	[135]
miR-760	Low expression in SW1990, AsPC-1, PANC-1, and BxPC-3 cells compared to normal pancreatic ductal epithelial cells	-	[136]
miR-1243	-	Venous invasion, a clinicopathological characteristic, is associated with the expression of miR-1243	[133]
miR-1285	Dropped in gemcitabine-resistant AsPC-1 and MIAPaCa-2 cells	-	[137]
miR-3656	Reduced in gemcitabine-resistant PANC-1 cells. Downregulated in cancer tissues compared to noncancerous tissues	Subcutaneous injections of PANC-1 cells overexpressing miR-3656 + intraperitoneal injections of gemcitabine (15 mg/kg). Poor patient prognosis is correlated with low miR-3656 levels	[138]

**Table 3:** CircRNA, lncRNA, and therapeutic resistance in PaC.

LncRNA	Expression	In Vivo Experiment and/or Clinical Relevance	Ref.
Circ-HIPK3	Abundant in gemcitabine-resistant cancer tissues	Poor overall survival of patients with high circ-HIPK3 expression	[126]
LINC00346	Highly expressed in cancer tissues as well as serum from patients	Intraperitoneal injections of gemcitabine (100 mg/kg) in mice bearing xenografts of LINC00346-depleted PANC-1 cells	[121,195]
LINC-DYNC2H1-4	Upregulated in gemcitabine-resistant BxPC-3 cells. Increased in cancer tissues compared to adjacent normal tissues	-	[118]
LncRNA-AB209630	Reduced in cancer tissues compared to adjacent normal controls	Poor patient prognosis is associated with low lncRNA-AB209630 levels	[194]
LncRNA-GAS5	Downregulated in gemcitabine-resistant SW1990 cells and 5-FU-resistnat PATU8988 cells	-	[46]
	Downregulated in cancer tissues compared to normal tissues	Intraperitoneal injections of gemcitabine (125 mg/kg) in mice bearing xenografts of lncRNA-GAS5-overexpressing PANC-1 cells. Intravenous injections of lncRNA-GAS5-overexpressing cells for metastasis analysis	[48]
LncRNA-GSTM3TV2	Upregulated in gemcitabine-resistant AsPC-1 and MIAPaCa-2 cells	Intraperitoneal injections of gemcitabine (25 mg/kg) in mice bearing xenografts of lncRNA-GSTM3TV2-overexpressing AsPC-1 cells. Poor survival rate of patients is associated with high expression of lncRNA-GSTM3TV2	[104]
LncRNA-HCP5	High expression is detected in gemcitabine-resistant SW1990 and PANC-1 cells. Upregulated in cancer tissues compared to normal tissues	Poor survival rate of patients is associated with high expression of lncRNA-HCP5	[125]
LncRNA-HOTTIP	Increased in cisplatin-resistant PANC-1, HS766T, and AsPC-1 cells	-	[115]
LncRNA-PVT1	Overexpressed in cancer tissues compared to adjacent pancreatic tissues	Intraperitoneal injections of gemcitabine (50 mg/kg) in mice bearing xenografts of PANC-1 cells stably expressing lncRNA- PVT1. Correlated with vascular infiltration and distant metastasis. Poor overall survival of patients with high lncRNA-PVT1 expression	[135,196]
LncRNA-SBF2-AS1	Abundantly expressed in gemcitabine-resistant AsPC-1 and PANC-1 cells. High expression is detected in cancer tissues compared to adjacent normal tissues	High expression is correlated with lymph node metastasis and poor overall survival of patients	[117]
LncRNA-SLC7A11-AS1	Highly expressed in gemcitabine-resistant BxPC-3 cells. Upregulated in cancer tissues compared to adjacent normal tissues	Intraperitoneal injections of gemcitabine (50 mg/kg) in mice bearing xenografts of lncRNA-SLC7A11-AS1-depleted PANC-1 cells. Negatively correlated with overall survival of patients	[197]
LncRNA-SNHG14	Higher in cancer tissues than normal tissues	-	[111]
LncRNA-TUG1	Overexpressed in several cell lines (PANC-1, PANC-28, BxPC-3, and SW1990) and cancer tissues	-	[198]

## Data Availability

Not applicable.
